# Purification of an Acidic Polysaccharide with Anticoagulant Activity from the Marine Sponge *Sarcotragus spinosulus*

**DOI:** 10.3390/md22030139

**Published:** 2024-03-21

**Authors:** Gabriele Nieddu, Gabriele Obino, Cristina Ciampelli, Antonio Brunetti, Tiziana Cubeddu, Renata Manconi, Giacinta Angela Stocchino, Giovanni Andrea Deiana, Marilena Formato, Antonio Junior Lepedda

**Affiliations:** 1 Department of Biomedical Sciences, University of Sassari, Viale San Pietro, 43b, 07100 Sassari, Italy; gobino@uniss.it (G.O.); c.ciampelli@phd.uniss.it (C.C.); brunetti@uniss.it (A.B.); formato@uniss.it (M.F.); 2 MERLN Institute for Technology-Inspired Regenerative Medicine, Complex Tissue Regeneration Department, Maastricht University, 6200 MD Maastricht, The Netherlands; 3 Department of Veterinary Medicine, University of Sassari, Via Vienna 2, 07100 Sassari, Italy; tcubeddu@uniss.it (T.C.); rmanconi@uniss.it (R.M.); giacistocchino@gmail.com (G.A.S.); 4 Department of Medicine, Surgery and Pharmacy, University of Sassari, Viale San Pietro, 43b, 07100 Sassari, Italy; gdeiana@uniss.it; 5 Struttura Complessa Laboratorio Unico di Analisi Cliniche Chimico-Ematologiche, Azienda Ospedaliera Universitaria, 07100 Sassari, Italy

**Keywords:** fucosylated chondroitin sulphate, marine invertebrates, anticoagulant activity, sustainable sponge culture, Porifera

## Abstract

Thromboembolic conditions are the most common cause of death in developed countries. Anticoagulant therapy is the treatment of choice, and heparinoids and warfarin are the most adopted drugs. Sulphated polysaccharides extracted from marine organisms have been demonstrated to be effective alternatives, blocking thrombus formation by inhibiting some factors involved in the coagulation cascade. In this study, four acidic glycan fractions from the marine sponge *Sarcotragus spinosulus* were purified by anion-exchange chromatography, and their anticoagulant properties were investigated through APTT and PT assays and compared with both standard glycosaminoglycans and holothurian sulphated polysaccharides. Moreover, their topographic localization was assessed through histological analysis, and their cytocompatibility was tested on a human fibroblast cell line. A positive correlation between the amount of acid glycans and the inhibitory effect towards both the intrinsic and extrinsic coagulation pathways was observed. The most effective anticoagulant activity was shown by a highly charged fraction, which accounted for almost half (about 40%) of the total hexuronate-containing polysaccharides. Its preliminary structural characterization, performed through infrared spectroscopy and nuclear magnetic resonance, suggested that it may consist of a fucosylated chondroitin sulphate, whose unique structure may be responsible for the anticoagulant activity reported herein for the first time.

## 1. Introduction

Coagulation, also known as clotting, is a dynamic biochemical process responsible for the healing and prevention of bleeding occurring after blood vessel injury [1]. It involves the activation of a series of coagulation factors interacting in a precise sequence to form a clot at the site of injury. This process is essential for the survival of organisms, as it prevents excessive blood loss and maintains blood vessel integrity [2]. The balance between factors that promote and inhibit the formation of the clot is called haemostasis, a term that literally means “stop of bleeding” and comes from the two ancient Greek words *haeme* and *stasis*, which stand for blood and to stop, respectively [3].

The first theories describing blood coagulation processes date back to the 1960s, when the hypotheses of two theories called the “waterfall” and “cascade” were proposed by Davie and Ratnoff [4], as well as by Macfarlane [5], explaining how a series of inactive enzymes can be activated one after the other, like a cascade, ultimately leading to the formation of a blood clot [6].

The coagulation process can be divided into three main pathways: the extrinsic, the intrinsic, and the common pathway. The first one is activated by tissue factor (TF), abundantly expressed by both adventitial cells surrounding blood vessels and subendothelial tissues [7] and exposed as a consequence of tissue damage, which binds with and activates factor VII [8], finally leading to the formation of a fibrin clot. On the other hand, the intrinsic pathway, also known as the contact pathway, is triggered by negatively charged surfaces coming into contact with factor XII, which is converted into its active conformation and can, in turn, activate factor XI and sequentially factor IX, which binds with factor VIII [9,10]. Both pathways converge on the common pathway by activating factor X, which forms a complex with its cofactor (factor V), tissue phospholipids, platelet phospholipids, and calcium, thus creating a stable clot [2].

All these events are tightly regulated to prevent excessive clotting, which can lead to life-threatening conditions such as deep vein thrombosis, pulmonary embolism, stroke, and other complications associated with vascular-related diseases, which represent the most common cause of death in developed countries [11,12,13]. At the same time, it is also important to maintain adequate clotting to prevent excessive bleeding, particularly during surgery or injury [14].

The identification of new natural molecules with anticoagulant activity is a key issue, as it can lead to the development of new drugs able to prevent excessive clotting without increasing the risk of bleeding and avoiding other side effects. Currently, the most widely used drugs for therapeutic purposes are warfarin, heparin (Hep), and Hep derivatives, whose ability to prevent excessive coagulation is effective but induces some considerable collateral effects, such as nausea, vomiting, abdominal pain, thrombocytopenia, significant haemorrhagic episodes, bruising, osteoporosis, and changes in lipid metabolism [15,16,17]. Furthermore, since clinically used Hep and its low-molecular-weight forms are commonly of porcine or bovine origin, some patients may not be prone to its use, for example, due to religious beliefs or nutritional habits. To overcome all these problems, alternative molecules purified from different sources have been tested so far, with promising results [9,18,19,20].

In this context, it has been widely reported that marine organisms are a rich source of bioactive compounds, potentially useful for various medical applications. Among them, sulphated polysaccharides show interesting biological properties similar to those reported for mammalian glycosaminoglycans (GAGs) [21], including anticoagulant [20], antimalarial [22], antitumoral [23], and anti-HIV-1 [24], as well as anti-inflammatory [25], activities. More in detail, anticoagulant properties have been reported for fucoidan purified from holothurians [26] and seaweeds [27,28], as well as for fucosylated chondroitin sulphate (FCS), which, as yet, has been found only in the holothurians’ body wall [29,30]. The latter, by selectively interfering with the intrinsic coagulation pathway with negligible bleeding risk if compared to heparinoids and warfarin, represents a very promising alternative to commonly used anticoagulant drugs [31].

Other marine invertebrates, such as Porifera, have been proven to produce a wide array of bioactive compounds that are potentially useful for both pharmaceutical and commercial applications [9,32,33,34,35]. 

A wide diversity of sulphated polysaccharides is synthesized by different species of marine sponges; their structural heterogeneity and complexity, concerning the molecular masses, the relative proportions of constituent sugars, and especially the sulphation degree, have been related to their biological role as cell adhesion factors involved in species-specific cell re-aggregation [36,37,38,39,40,41,42,43]. 

The usefulness of sponge acid polysaccharides for medical applications in the context of cardiovascular diseases has not been investigated so far.

This study focused on the proteolytic extraction of acidic glycans from the marine sponge *Sarcotragus spinosulus*, their purification by anion-exchange chromatography, and the evaluation of their anticoagulant activity. Furthermore, their distribution in the extracellular matrix (ECM) was determined by histological analysis, and their cytocompatibility was determined on a human skin fibroblast cell line. 

Corresponding polysaccharide fractions were obtained by the same purification procedures from the body wall of *Holothuria tubulosa*, one of the most abundant Mediterranean sea cucumber species, and were used, in this study, as a positive control for coagulation assays, since it has been widely demonstrated that some hexuronate-containing glycans, particularly sulphated fucoidans [44] and FCSs [45,46] extracted from several holothurian species, represent efficient inhibitors of the coagulation cascade. The structural characterization of the sponge acidic glycan fraction with the highest anticoagulant potential was assessed via FT-IR and NMR.

## 2. Results and Discussion

All data collected for *Sarcotragus spinosulus* were compared with those obtained from the marine invertebrate *Holothuria tubulosa*, for which the presence of acidic polysaccharides able to interfere with the coagulation cascade has been reported by various studies [19,26].

### 2.1. Histological Analysis

#### 2.1.1. *Sarcotragus spinosulus*

The sponge body, lined by the epidermis, was characterized by two districts, i.e., an outer district (ectosome) rich in canals and cavities (aquiferous system) and an inner district (endosome/choanosome) with choanocyte chambers, cavities, and canals. Both districts were filled by extracellular matrix (ECM) harbouring different types of isolated cells. The ECM layout was supported by densely structured collagenic skeletal networks (filamentous and fibrous) and scanty exogenous mineral structures.

**Figure 1:**
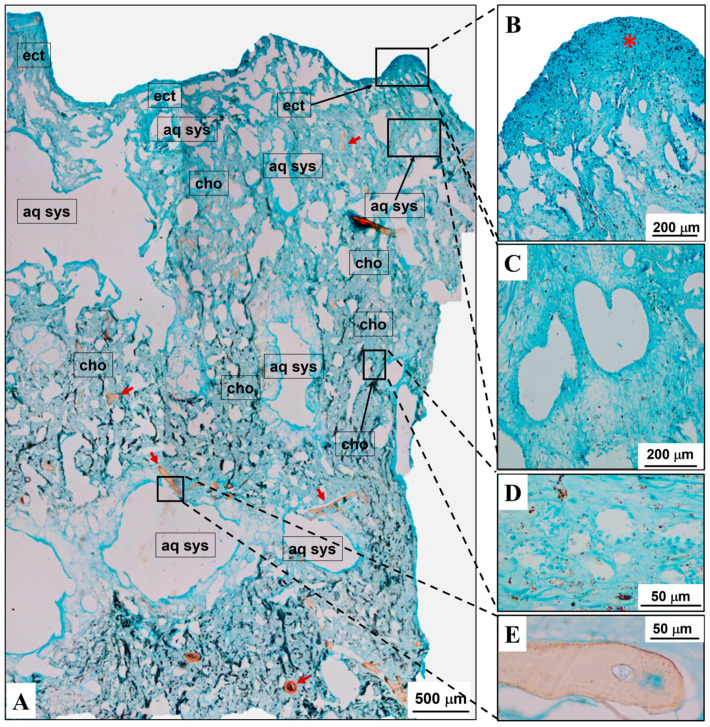
*Sarcotragus spinosulus* (Porifera, Irciniidae) as a model of body plan architecture in a marine basal Metazoa. (**A**) Acidic glycans’ topographic distribution (evidenced by Alcian blue staining) in the ubiquitous ECM is highlighted as a range of light blue to blue-turquoise shades at the level of both outer (ectosome, namely ect) and inner (endosome/choanosome, namely cho) body districts. (**B**) Outer body district (ectosome) has a dermal membrane compact layout (intensely blue-turquoise, red asterisk) and the less-coloured underlying layer is rich in canals and cavities (inner ectosome). Cells with nuclei are pinkish spots. (**C**) Intensely blue-turquoise shades show the aquiferous system (aq sys) outlines (cavities and canals, endosome/choanosome) surrounding a less-coloured ECM. Cells with nuclei are pinkish spots. (**D**) Choanocyte chambers within a light blue ECM (endosome/choanosome). (**E**) Acidic glycans within the skeletal fibres (red arrow).

The ECM staining with Alcian blue highlighted varying intensities of blue-turquoise in both body districts, where cell nuclei were pinkish. In particular, (i) the subepidermis area and (ii) the outlines of cavities and canals (aquiferous system) were intensely blue-turquoise (Figure 1). At the inner district (endosome/choanosome), the ECM was blue-turquoise as well but was somewhat less intensely coloured than that of the ectosomal district (Figure 1A,C). The skeletal networks were made of light blue to uncoloured filaments and light pinkish to uncoloured fibres, the latter sometimes with light blue-turquoise areas (Figure 1A,E). 

These results suggest that the ECM of *S. spinosulus* is particularly rich in acidic glycans, confirming data from the literature [47]. Although sponges are the most basal and oldest metazoans, their ECM, similarly to that of higher taxa, is mostly composed of collagen and proteoglycan-like molecules, together with minor amounts of structural proteins. According to the recent literature, the poriferan polysaccharidic chains of the proteoglycan-like molecules differ from classical mammalian glycosaminoglycans [42,48].

#### 2.1.2. *Holothuria tubulosa*

**Figure 2:**
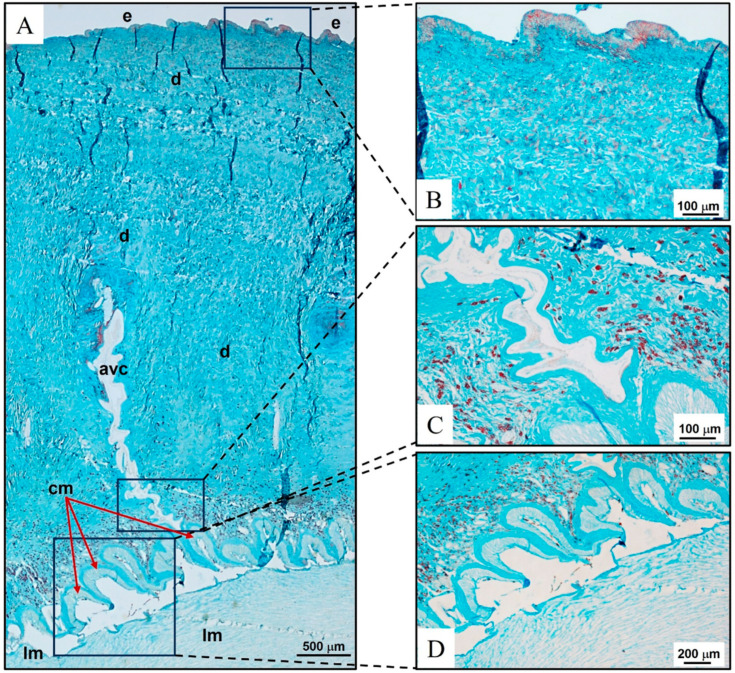
*Holothuria tubulosa* (Echinodermata, Holothuriidae) as a model of body wall architecture in a complex marine invertebrate (Metazoa). (**A**) Architecture of the body wall district. Compact dermis with extremely abundant ECM occupying most of the wall’s thickness. (**B**) Intensely blue-turquoise-coloured thin acellular cuticle and dermis on staining with Alcian blue. The epidermis was less intensely coloured. (**C**) Outline of a pedicellar canal (aquiferous vascular system) is more coloured than the surrounding body wall dermis ECM. (**D**) Circular muscles supported by a compact, coat-like ECM are more coloured than the surrounding dermis. A light blue-turquoise stain is evident in circular and longitudinal muscles. Epidermidis (e), dermis (d), aquiferous vascular system (avc), circular muscles (cm), longitudinal muscles (lm).

The sea cucumber body wall district was characterized by (i) an epidermis covered by a thin acellular cuticle and (ii) a dermis connective tissue with few cells embedded in an extremely abundant ECM occupying most of the wall’s thickness (Figure 2A). Below the dermis, two muscular layers internally lined the perivisceral coelom (body cavity), i.e., a circular muscle layer and a layer composed of five pairs of longitudinal muscles (Figure 2A,D). The body wall was crossed by the pedicellar canals of the aquiferous vascular system (Figure 2A,C). The cuticle and dermis were intensely blue-turquoise coloured on staining with Alcian blue (Figure 2). The epidermis was less intensely coloured (Figure 2B). The ECM delimiting the aquiferous pedicellar canals was slightly more coloured than the surrounding dermis ECM (Figure 2A,C). Also, the ECM compact layer supporting the circular muscles was more coloured than the surrounding dermis (Figure 2A,D). A light blue turquoise stain was evident within the circular and longitudinal muscles (Figure 2C,D).

The abundant presence of polysaccharides in the ECM of sea cucumber body wall previously reported [49,50] has been confirmed in the present study, as evidenced by the intense blue-turquoise staining, indicative of a high amount of acidic glycans in this body district of holothurians.

### 2.2. Purification and Biochemical Analyses

Following papain extraction from the ECM of both species, four polysaccharide fractions were obtained through anion-exchange chromatography by increasing the ionic strength stepwise (0.5 M, 1.0 M, 1.5 M, and 2.0 M LiCl concentration). Each fraction was analysed for its hexuronate content and subjected to qualitative analysis using electrophoretic techniques. For the sake of clarity, each of the fractions purified from both marine invertebrates was assigned an acronym composed of the initials of the systematic name and number, from 1 to 4, corresponding to the sequential elution order.

#### 2.2.1. Quantitative Analysis

All purified fractions were quantified using the carbazole assay as described beyond, yielding positive results for each fraction eluted from all specimens. As reported in Table 1, the total yield of hexuronate-containing glycans isolated from *S. spinosulus* was 2.234 mg/g dry weight. Concurrently, considerable variability in the content of uronic acid (UA) was registered for each fraction purified. In particular, the most abundant glycan fractions are represented by Ss3 (43.78%) and Ss1 (40.15%).

On the other hand, the total amount of hexuronate-containing glycans extracted from the holothurian body wall was more than double the amount from sponge samples and mainly represented by the Ht3 fraction (86.82%). 

Overall, quantitative data indicated that the distribution of hexuronate-containing polysaccharides in the *S. spinosulus* fractions was different compared to *H. tubulosa* fractions, with the high-charge and low-charge molecular species being in similar proportions (Table 1).

#### 2.2.2. Electrophoretic Profiles

Preliminary structural characterisation of hexuronate-containing glycan fractions was performed through comparative analysis of electrophoretic profiles on a polyacrylamide gel.

Carbohydrate electrophoresis (C-PAGE) allows for the separation of polysaccharides based on their molecular masses; moreover, the treatment with the cationic carbocyanine dye Stains-all stains polysaccharides with specific colours, which turn from blue to purple to yellow as the degree of sulphation increases [51].

**Figure 3:**
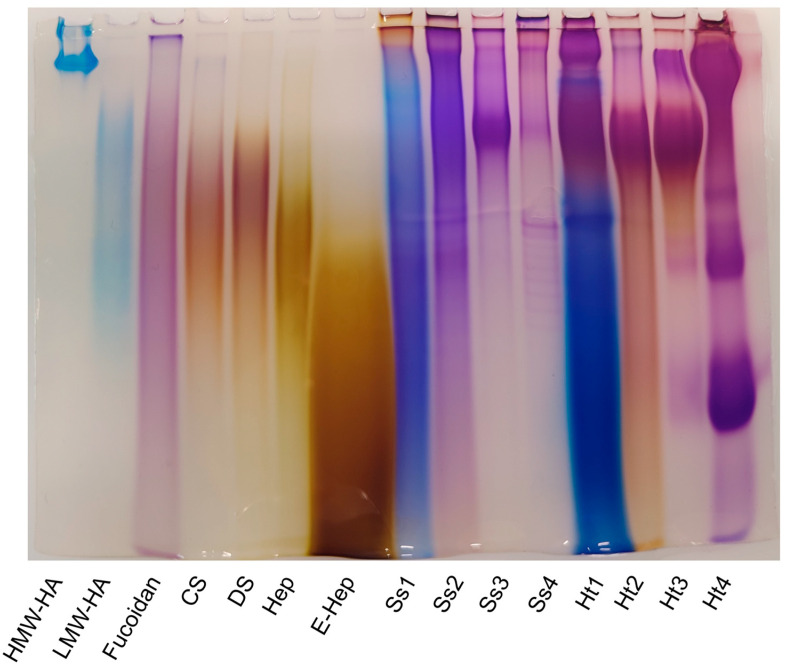
Electrophoretic profiles of standard polysaccharides (high-molecular-weight hyaluronic acid (HMW-HA), low-molecular-weight hyaluronic acid (LMW-HA), fucoidan, chondroitin sulphate (CS), dermatan sulphate (DS), heparin (Hep), and enoxaparin (E-Hep)) and purified fractions from *Sarcotragus spinosulus* (Ss1–Ss4) and *Holothuria tubulosa* (Ht1–Ht4).

All acidic glycan fractions purified from both marine invertebrates consisted of a wide range of molecular weight polysaccharides; each lane was somewhat smeared, just as is evident for standard polysaccharides (Figure 3).

Moreover, thanks to the metachromasia phenomenon caused by the dye used, it could be appreciated that different eluted fractions exhibited different degrees of sulphation, which increased together with the ionic strength of the elution buffer used. 

By comparing the colours of sponge fractions lanes with those of standard glycans, it was possible to perceive that Ss1 showed a colour shade between that of the non-sulphated high- and low-molecular-weight hyaluronic acid and fucoidan, thus suggesting a very low sulphation degree. Moreover, fraction Ss2 showed a profile similar to the fucoidan standard, while Ss3 and Ss4 exhibited a more purplish colour with respect to fucoidan but less yellowish than chondroitin sulphate (CS), dermatan sulphate (DS), heparin (Hep), and enoxaparin (E-Hep), suggesting that they were composed of less sulphated glycans compared to standard glycosaminoglycans. Overall, by comparing corresponding fractions obtained from *S. spinosulus* and *H. tubulosa*, we detected similar patterns for Ss3 and Ht3 only, and the holothurian polysaccharide was considerably more sulphated.

#### 2.2.3. In Vitro Cytocompatibility Assessment

A preliminary evaluation of the cytocompatibility of each hexuronate-containing glycans fraction purified from *S. spinosulus* was performed herein, for the first time, on human fibroblasts, well known to be a model for cell proliferation and adhesion studies to evaluate their potential side effects on cellular metabolic activity. Reduction potential, positively correlated with cell viability, was evaluated by using PrestoBlue™ reagent (Waltham, MA, USA) in triplicate for each condition at each timepoint. 

**Figure 4:**
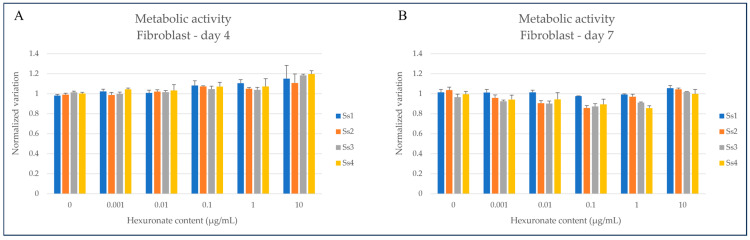
Metabolic activity of human fibroblast cell line incubated with purified fractions from *Sarcotragus spinosulus*, measured after 4 and 7 days (panels (**A**) and (**B**), respectively). Each eluate was tested at different concentrations, ranging from 0.001 µg/mL to 10 µg/mL, and fluorescence was measured after incubation with PrestoBlue™ reagent.

Overall, the obtained data showed that all hexuronate-containing fractions purified from *S. spinosulus* did not have remarkable effects on cell viability or proliferation, as no significant changes in reducing potential were observed for any of the assayed concentrations, after 96 and 168 h of incubation, with respect to untreated controls (Figure 4).

### 2.3. Anticoagulant Activity of Purified Glycan Fractions

The acidic glycans purified from *S. spinosulus* were herein assessed for their potential activity, in vitro, towards the coagulation cascade. The ability of purified fractions from *S. spinosulus* to prolong the clotting time for both the intrinsic and extrinsic pathway of the coagulation cascade was evaluated using activated partial thromboplastin time (APTT) and prothrombin time (PT) assays, respectively, and compared with standard glycosaminoglycans (GAGs) (Hep, E-Hep, CS + DS, and CS) and *H. tubulosa* fractions, assuming both Hep and E-Hep as positive controls. 

#### 2.3.1. Inhibition of the Intrinsic Pathway of Coagulation

**Figure 5:**
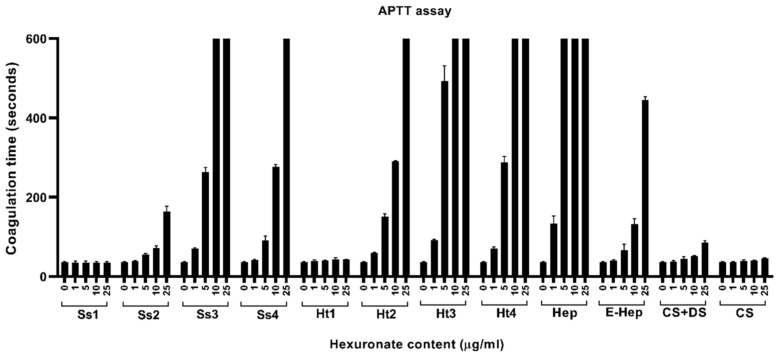
In vitro anticoagulant activity on intrinsic pathway of acidic glycan fractions purified from *Sarcotragus spinosulus* (Ss1–Ss4) and *Holothuria tubulosa* (Ht1–Ht4) and standard GAGs. Values are reported as mean ± SD.

Figure 5 reports the effects of purified fractions from *S. spinosulus* (Ss1–Ss4), compared to both standard GAGs and *H. tubulosa* (Ht1–Ht4) glycans, on the intrinsic cascade of coagulation. As expected, data concerning standard GAGs were consistent with the literature, confirming that Hep, together with its low-molecular-weight fragments, were the most effective compounds able to inhibit clot formation [52], whereas low or no effects were evidenced for both CS + DS and CS, as previously described [53]. Regarding the four sponge fractions, no activity was evidenced for the first one, whereas Ss2, Ss3, and Ss4 showed variable inhibitory effects. More in detail, the Ss2 fraction showed a limited effect at 25 µg/mL, whereas Ss3 and Ss4 fractions were found to be the most effective inhibitors of the intrinsic pathway at 10–25 µg/mL and 25 µg/mL, respectively, with results between those recorded for Hep and E-Hep. By comparing each of the fractions obtained from the two species, those from holothurian samples were more effective in inhibiting coagulation. Indeed, Ht2, Ht3, and Ht4 had remarkable effects, as their inhibitory potential was higher than that of E-Hep although lower than unfractionated Hep, in agreement with the data reported elsewhere [19,26]. 

As far as we know, the obtained data indicated, for the first time, that acidic polysaccharides from marine sponges, similar to those extracted from sea cucumbers, can interfere selectively with the contact pathways, even though it is with a lower activity compared to Hep.

#### 2.3.2. Inhibition of the Extrinsic Pathway of Coagulation

**Figure 6:**
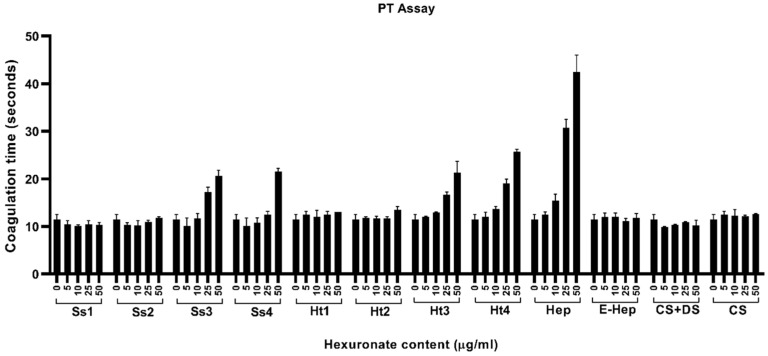
In vitro anticoagulant activity on extrinsic pathway of acidic glycan fractions purified from *Sarcotragus spinosulus* (Ss1–Ss4) and *Holothuria tubulosa* (Ht1–Ht4) and standard GAGs. Values are reported as mean ± SD.

Figure 6 reports the effects of both standard GAGs and purified fractions from sponge (Ss1–Ss4) and holothurian (Ht1–Ht4) on the extrinsic cascade of coagulation. Among the standard GAGs, only Hep showed inhibitory activity, whereas, as expected, no significant effects on the PT assay were evidenced for E-Hep due to the low concentrations used, as indicated by the manufacturer and stated elsewhere [54], and for CS + DS and CS. With regard to the purified fractions, only Ss3 and Ss4 were found to be able to slightly extend the PT, with an activity comparable to that observed for Ht3 and Ht4, in agreement with previous papers reporting that holothurians’ most negatively charged fractions have an inhibitory effect, although reduced with respect to Hep, on the extrinsic pathway [55,56,57]. 

All these results were consistent with the literature, indicating that sea cucumbers contain FCS with strong anticoagulant activity, particularly on the intrinsic pathway [58]. The anticoagulant properties that we have described for some purified fractions from *S. spinosulus* may be due to peculiar structural features including saccharide composition, chain length, sulphation pattern, and glycosidic bonds.

Since the fraction that demonstrated to be more effective in inhibiting the formation of the clot, towards both the intrinsic and extrinsic pathway, was Ss3, we decided to elucidate its structure by means of spectroscopic analyses.

### 2.4. Structural Characterization via FT-IR and NMR Spectroscopy

#### 2.4.1. Fourier Transform Infrared (FT-IR) Spectroscopy

**Figure 7:**
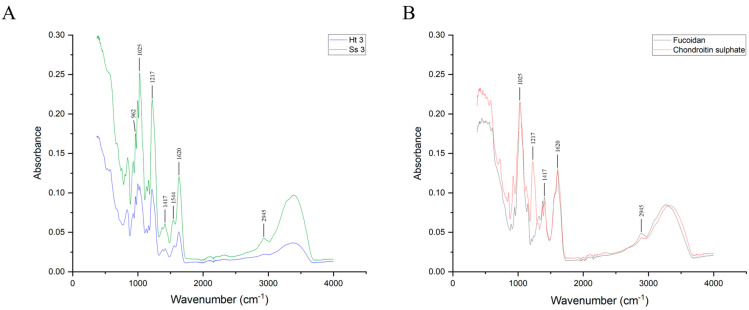
FT-IR spectra of Ss3 (green) and Ht3 (blue) fractions of *Sarcotragus spinosulus* and *Holothuria tubulosa* (panel (**A**)) and of standard fucoidan (black) and CS (red) (panel (**B**)).

FT-IR spectra of the fractions showing the most remarkable anticoagulant activity from both *S. spinosulus* and *H. tubulosa*, and of CS and fucoidan standards, were collected between 400 cm^−1^ and 4000 cm^−1^, as reported in Figure 7A,B. The different signals obtained from standard polysaccharides and the purified fractions showed a high percentage of matching. In fact, a significant absorbance at around 570 cm^−1^, 1025 cm^−1^, 1220 cm^−1^, and 1370 cm^−1^ was detected for every sample, corresponding, respectively, to S-O stretching vibration [59], C-O and C-C stretching vibrations of the pyranose ring, demonstrated to be common to all polysaccharides [60,61], symmetric stretching of S=O [62], and uronic acid O-C=O bending or, alternatively, CH_3_ group bending of L-fucopyranosyl [63,64]; all these signals are distinctive of polysaccharides, thus proving that the adopted method was effective for the purification of acidic glycans from different sources.

Signals at around 720 cm^−1^, 930 cm^−1^, 1130 cm^−1^, 1160 cm^−1^, 1417 cm^−1^, and 2945 cm^−1^ were collected, corresponding to C-H bending, to the presence of 3,6-anhydro-D-galactose [60], to C-C-C symmetric vibrations of glycosidic linkage cycles [26], to a C-O-C glycosidic linkage group [64], to O-C=O uronic acid presence [26], and to C-H stretching vibrations [56], respectively. All these characteristic absorptions suggest that the backbone of the glycans present in the third fraction of both *S. spinosulus* and *H. tubulosa* is similar to CS since they all contain glucuronic acid and that the differences observed may be due to the presence of fucosyl branches in Ss3 and Ht3 polysaccharides, as suggested by the signal detected at 962 cm^−1^, corresponding to both asymmetric and symmetric vibrations of methenyl groups in fucose residues, as reported by previous studies [65]. 

As highlighted by some signals recorded at 677 cm^−1^, as well as in the region of 800–840 cm^−1^, the third fraction from both *S. spinosulus* and *H. tubulosa* contains polysaccharides with a high degree of sulphation, in addition to containing a secondary amine, as evidenced by the signal recorded at 1544 cm^−1^ and 1555 cm^−1^, for Ss3 and Ht3, respectively, corresponding to C-N vibrations of the N-acetyl group [64].

Overall, the analysis of acquired spectra, corroborated by both FT-IR and NMR data from the literature, allowed us to determine characteristic absorption bands, likely corresponding to the FCS polysaccharide chain, consisting of the repeating trisaccharide unit N-Acetyl-D-glucosamine and D-glucuronic acid backbone branched with L-Fucose, previously reported to be exclusive to the body wall of several sea cucumber species. Therefore, FT-IR data suggest the presence of the branched acidic glycan FCS in the complex three-dimensional dynamic network that surrounds and provides structural support to sponge cells.

#### 2.4.2. Nuclear Magnetic Resonance (NMR) Analysis

**Figure 8:**
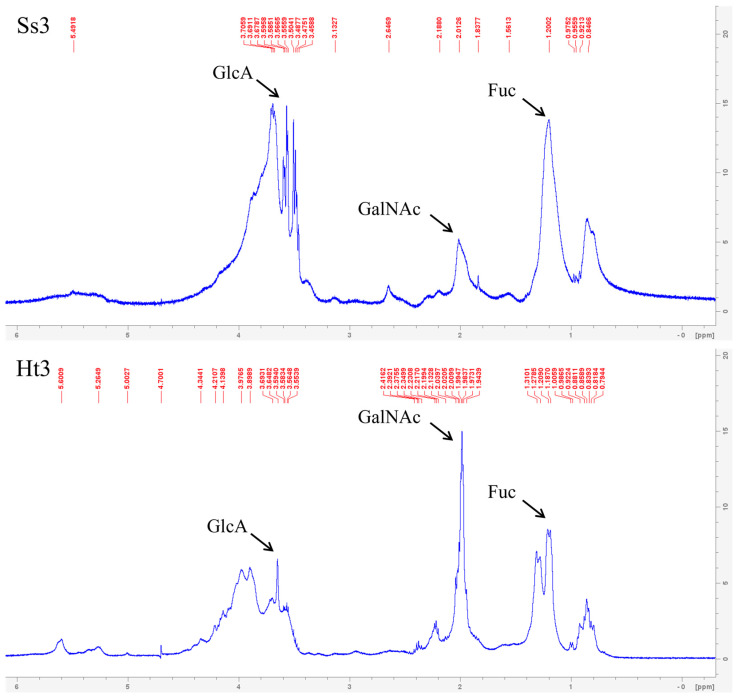
^1^H NMR spectra of fucosylated chondroitin sulphates from Ss3 (*Sarcotragus spinosulus*) and Ht3 (*Holothuria tubulosa*).

A preliminary analysis was conducted by proton (^1^H) nuclear magnetic resonance (NMR). Recorded signals indicated that the purified fractions Ss3 and Ht3 showed characteristic signals of fucose (Fuc), N-Acetylgalactosamine (GalNAc), and glucuronic acid (GlcA) (Figure 8).

More in detail, signals recorded at δ 1.21 ppm were assigned to unsulphated Fuc [66,67], at δ 2.00 ppm to GalNAc [67,68,69], and at δ 3.59 to non-substituted GlcA residues [66].

Moreover, the Ss3 spectrum presented a further peak at δ 3.39, corresponding to H-2 of the non-substituted GlcA residues [66], thus confirming the presence of hexuronic acid in the structure of this polysaccharide fraction.

No information on the branching, sulphation degree, or molar ratio of monosaccharides was obtained from the structural analyses performed. Although data recorded by NMR confirmed results obtained by infrared spectroscopy, indicating that Ss3 and Ht3 polysaccharides share common structural features, these findings deserve further confirmation due to the great heterogeneity and complexity described for poriferan ECM polysaccharides.

Species-specific variations in both chemical composition and molecular masses of sulphated polysaccharides have been reported [37,42]. This wide structural diversity probably reflects their pivotal role in sponge ECM organization and in the well-known sponge adaptive strategies, such as cell totipotence, cell dedifferentiation, chronic morphogenesis, and modular/clonal organization of the body plan [36,38,39,40,41,43].

## 3. Materials and Methods

### 3.1. Chemicals and Equipment

High-molecular-weight hyaluronic acid (HMW-HA) sodium salt from *Streptococcus equi* (1,500,000–1,750,000 Da), low-molecular-weight hyaluronic acid (LMW-HA) sodium salt from *Streptococcus equi* (8000–15,000 Da), chondroitin sulphate (CS) sodium salt from bovine trachea, dermatan sulphate (DS) from porcine intestinal mucosa, heparan sulphate (HS) sodium salt from bovine kidney, heparin (Hep) sodium salt from porcine intestinal mucosa, fucoidan from marine brown algae *Macrocystis pyrifera*, glycerol, Stains-all, cresol red, deuterium oxide, trizma base (Tris), lithium chloride, sodium acetate, acrylamide/bis-acrylamide 30% solution (29:1), alcian blue 8GX, diethylaminoethyl-(DEAE)Sephacel, ethylenediaminetetraacetic acid (EDTA), L-cysteine, neutral red, and glucuronolactone were purchased from Merck (Merck KGaA, Darmstadt, Germany). Ethanol was bought from Honeywell International Inc. (Charlotte, NC, USA). Papain, sulphuric acid, magnesium chloride, Dulbecco’s Modified Eagle Medium (DMEM) high glucose, GlutaMAX™ Supplement, Fetal Bovine Serum (FBS), phosphate-buffered saline (PBS), and PrestoBlue™ Cell Viability Reagent were acquired from Themo Fisher Scientific Inc. (Waltham, MA, USA). A low-molecular-weight heparin (E-Hep), namely enoxaparin, with the trade name Clexane^®^, was purchased from Sanofi (Paris, France), while activated partial thromboplastin time (APTT) (Dade^®^ Actin^®^ FSL) and prothrombin time (PT) (Dade^®^ Innovin^®^) assay kits were purchased from Siemens Healthcare GMBH (Erlangen, Germany). Coagulation control plasma was from Biolab Diagnostics (Sant’Antonio Abate, Italy). Formic acid, formalin, acetone, sodium tetraborate decahydrate, carbazole, and acetic acid were from Carlo Erba Reagents GMBH (Emmendingen, Germany). The human fibroblast (CRL-2522 ATCC^®^) cell line was collected from the American Type Culture Collection (Rockville, MD, USA). Amicon Ultra-15 Centrifugal Filter Units were produced by Millipore (Burlington, MA, USA). Xylene and paraffin were from Bio-Optica Milano spa (Milano, Italy). Econo-Column chromatography columns, the GS-800 calibrated densitometer, and the Mini Protean II cell vertical slab gel electrophoresis apparatus were acquired from Bio-Rad laboratories (Hercules, CA, USA). A VictorX5 Multimode Plate Reader was bought from PerkinElmer (Waltham, MA, USA). The Bruker alpha compact FT-IR spectrometer and Bruker Avance III 400 MHz NMR spectrometer were from Bruker (Karlsruhe, Germany). The Edwards Modulyo Freeze Dryer was from Edwards Vacuum (Burgess Hill, UK). A Nikon ECLIPSE 80i Light Microscope, Nikon Digital-Sight DS-FI camera, and Nikon D3100 reflex camera were acquired from Nikon Corporation (Tokyo, Japan).

### 3.2. Experimental Models

*Sarcotragus spinosulus* Schmidt, 1862 (Porifera, Dictyoceratida, Irciniidae) is a sessile, benthic, basal Metazoa with no organs and true tissues. It is a photophilous demosponge inhabiting Mediterranean shallow waters, with an asymmetric, massive, rounded growth form. Its body surface, brownish in colour, black to dark grey in vivo, is irregularly conulose and entirely covered by a dermal membrane; the interior is orange to light brown. The three-dimensional collagenic (horny) endoskeleton with a complex architecture is embedded in the amorphous jelly-like extracellular matrix (ECM) of the entire sponge body [47].

*Holothuria tubulosa* Gmelin, 1791 (Echinodermata, Holothuroidea, Holothuriidae) is a benthic, sedentary, complex Metazoa living in the Mediterranean Sea (surface to 100 m depth). Its bilateral body is roughly cylindrical along the oro-aboral axis and dark brown, with numerous conical papillae [70]. The body wall of connective tissue, rich in ECM, with embedded calcareous skeletal ossicles, encircles the coelom in which internal organs are localized. 

### 3.3. Sponge and Holothurian Sample Harvesting

Reared specimens of *S. spinosulus* were collected from a sustainable, shallow water experimental sponge culture plant [71,72] harboured in the Tramariglio Cove (40°35′32.47″ N 8°10′11.50″ E, Capo Caccia—Isola Piana Marine Protected Area, Northern Sardinian Sea, Western Mediterranean). 

Wild specimens of *H. tubulosa* were collected along the Porto Torres coast (40°50′17.781″ N 8°24′33.955″ E, Asinara Gulf, Northern Sardinian Sea, Western Mediterranean). 

All specimens of sponges and holothurians were immediately kept in seawater in refrigerated bags and transferred to the Sassari University laboratories.

### 3.4. Histological Analyses

Representative body fragments of *S. spinosulus* were dissected, after hypothermia, using a scalpel. 

For downstream histological evaluation, samples were fixed in formalin 10% at room temperature before being fully dehydrated in ascending ethanol series, cleared in xylene, and embedded in paraffin. 

Specimens of *H. tubulosa* were killed by hypothermia, and body wall fragments were fixed in formalin 10% at room temperature, rinsed in distilled water, and decalcified in formic acid 8% for 24 h to remove skeletal ossicles. After decalcification, samples were rinsed in distilled water and then dehydrated in an ascending ethanol series, cleared in xylene, and embedded in paraffin.

Sponges and holothurian samples were sectioned at intervals of 3 µm and stained with Alcian blue (pH 2.5) to verify the presence and topographic distribution of acidic glycans and counterstained with neutral red to detect the nuclei of cells. Slides were examined under a Nikon ECLIPSE 80i Light Microscope, and microphotographs were taken with a Nikon Digital-Sight DS-FI camera.

### 3.5. Acidic Glycan Purification

Body fragments (*n* = 18) of *S. spinosulus* and body wall fragments (*n* = 7) of *H. tubulosa* were put in absolute ethanol for 120 h, at 4 °C, and then finely minced, before being incubated in acetone for 24 h in order to achieve complete dehydration and delipidation. Samples were centrifuged at 5000× *g* for 15 min, and the supernatant was discarded. After complete acetone evaporation, dehydrated and delipidated tissue (DDT) was rehydrated with 0.1 M sodium acetate buffer, pH 6.0, containing 5 mM EDTA, and 5 mM cysteine (15 mL per gram of tissue), at 4 °C for 24 h. Proteolytic treatment was performed by adding 1 U of papain per mg of DDT at 56 °C for 48 h; enzyme activity was stopped by boiling the mixture at 100 °C for 5 min. After centrifugation at 5000× *g* for 10 min, the supernatant was recovered and immediately loaded into a chromatography column packed with DEAE-Sephacel anion-exchange resin (10 mL of resin per gram of digested tissue), previously equilibrated with 50 mM sodium acetate, pH 6.0. The same buffer was used to wash the column until absorbance, measured at 280 nm, was less than 0.05. Acidic glycans were fractionated by performing four separate elution steps using 20 mM Tris-HCl buffer, pH 8.6, containing, alternatively, 0.5 M, 1 M, 1.5 M, and 2 M lithium chloride. Eluates were concentrated and dialysed against deionized water by means of Amicon Ultra-15 Centrifugal Filter Units with a 3 kDa cut-off, according to the manufacturer’s instructions. The following acronyms have been assigned to each purified fraction to indicate the four different elutions: 0.5 M fraction from *S. spinosulus* and *H. tubulosa*, Ss1 and Ht1, respectively; 1 M fraction from *S. spinosulus* and *H. tubulosa*, Ss2 and Ht2, respectively; 1.5 M fraction from *S. spinosulus* and *H. tubulosa*, Ss3 and Ht3, respectively; and 2 M fraction from *S. spinosulus* and *H. tubulosa*, Ss4 and Ht4, respectively. All samples were assayed in duplicate.

### 3.6. Hexuronic Acid Quantification

Each eluate was assayed for uronic acid (UA) content using the method of Bitter and Muir [73] with glucuronolactone as the standard, as reported elsewhere [74]. Briefly, 250 µL of either standard (from 5 to 40 µg UA/mL) or eluate were added with 1.25 mL of 25 mM sodium tetraborate decahydrate in concentrated sulphuric acid and incubated at 85 °C for 10 min. Afterwards, 50 µL of carbazole was added and, following 15 min of boiling, absorbance was read at 530 nm.

### 3.7. C-PAGE

Carbohydrate electrophoresis [51] was carried out in a Mini Protean II cell vertical slab gel electrophoresis apparatus, using 13.5% T, 3% C polyacrylamide running gels, overlaid with 5% T, 3% C stacking gel, using 40 mM acetic acid, and 40 mM Tris-HCl, pH 7.8, solution as the running buffer. A volume corresponding to 2.5 μg, in terms of UA, of each standard polysaccharide and glycan fraction was freeze-dried and then resolubilized in 10 μL of 62.5 mM Tris-HCl, pH 7.8, 10% glycerol, and 0.002% cresol red and loaded into the wells. The run was performed at 50 V for the first 10 min and then at 120 V until the dye front reached the bottom of the gel. Staining was performed by incubating every gel with 50 mL of a solution of 50% ethanol containing 0.005% Stains-all dye overnight and in the dark at room temperature. Gels were rehydrated with distilled water before being photographed with a Nikon D3100 reflex camera and acquired using a GS-800 calibrated densitometer.

### 3.8. Cell Culture and Metabolic Activity Assay

The cytocompatibility of all fractions of acidic glycans purified from *S. spinosulus* was assessed, in vitro, on a human skin fibroblasts cell line (ATCC, CRL-2522) to preliminarily evaluate the biological effects of these compounds. More in detail, metabolic activity was evaluated by means of PrestoBlue™ Cell Viability Reagent, according to the manufacturer’s instructions. Briefly, 5 × 10^4^ cells/well were suspended in 90 μL of DMEM added with 10% FBS, seeded in a 96-well plate, and treated with 10 μL of complete culture medium containing different polysaccharide final concentrations (0.001, 0.01, 0.1, 1, and 10 μg/mL, expressed as UA content). Untreated cells were used as controls.

At every time point, 11 μL of PrestoBlue™ was added to each well, and well plates were incubated at 37 °C and 5% CO_2_. After 45 min, the solution of PrestoBlue™/DMEM + 10% FBS was transferred into a new well plate, and fluorescence was measured with the excitation/emission wavelengths set at 540/590 nm, with VictorX5 multilabel counter. Cells were rinsed with PBS, and 100 µL of new culture media containing 10% FBS, together with the abovementioned concentrations of acidic glycans, was added to each well. Experiments were performed in triplicate.

### 3.9. Coagulation Assays

The anticoagulant activity of the four eluted fractions from *S. spinosulus* was evaluated in vitro and compared with that of *H. tubulosa* purified glycans, as well as standard glycosaminoglycans (GAGs), including Hep and E-Hep, as well as CS and DS, using commercially available kits by Siemens Healthcare. In particular, activated partial thromboplastin time (APTT) (Dade^®^ Actin^®^ FSL) and prothrombin time (PT) (Dade^®^ Innovin^®^) assays were performed to evaluate the inhibition of the intrinsic and extrinsic coagulation pathways, respectively. Commercial human coagulation control plasma was used for all coagulation tests.

Four dilutions of each sample were prepared and tested in order to assess the effects of these polysaccharides on the inhibition of clot formation. In particular, the APTT assay was performed with the following concentrations of either standard GAGs or purified glycans: 1 µg/mL, 5 µg/mL, 10 µg/mL, and 25 µg/mL, while the impact on the extrinsic pathway of the coagulation cascade was assayed for concentrations of 5 µg/mL, 10 µg/mL, 25 µg/mL, and 50 µg/mL.

The APTT assay was carried out according to the manufacturer’s guidelines. Briefly, 100 μL of plasma, previously added with purified polysaccharides or standard GAGs, was mixed with 100 μL of prewarmed FSL reagent and incubated at 37 °C for 3 min; then, 100 μL of a prewarmed 25 mM calcium chloride solution was added, and the resulting fibrin clot formation time was measured by optical detection. 

The PT assay was performed following the producer’s instructions as well. More in detail, 100 μL of plasma, containing purified acidic glycans or standard GAGs, was incubated at 37 °C for 1 min, and 200 μL of prewarmed Dade^®^ Innovin^®^ Reagent was added. Clot formation time was measured by optical detection.

All measurements were conducted in triplicate, and the results were reported as mean and standard deviation.

### 3.10. Fourier Transform Infrared (FT-IR) Spectroscopy

Hexuronate-containing fractions with remarkable anticoagulant properties, as well as standard fucoidan and CS, were freeze-dried overnight, followed by Fourier transform infrared spectroscopy (FT-IR) analyses. Spectra were collected using a Bruker Alpha spectrometer, with 128 scans at a spectral resolution of 2 cm^−1^.

All measurements were performed in the range of 400–4000 cm^−1^ at room temperature. Data were acquired and processed by Bruker Opus software, release 8.7 (Billerica, MA, USA).

### 3.11. Nuclear Magnetic Resonance (NMR) Spectroscopy

NMR spectra were recorded on a Bruker Avance III 400 MHz spectrometer (^1^H NMR: 400.13 MHz), (Billerica, MA, USA). A volume corresponding to 10 mg of both Ss3 and Ht3 fractions was freeze-dried and dissolved in 650 µL of 99.9% D_2_O. Measurements were performed at 298 K, with HOD suppression by pre-saturation. Signals were analysed by TopSpin 4.3.0 software (Bruker, Billerica, MA, USA).

## 4. Conclusions

In this study, four acidic polysaccharide fractions purified from the marine sponge *Sarcotragus spinosulus* were analysed to evaluate their biological activity towards the coagulation pathway, in addition to determining their localization and abundance in the extracellular matrix (ECM), as well as assessing their cytocompatibility in vitro. Histological analyses on representative sponge samples allowed us to confirm that Porifera ECM is rich in acidic glycans. 

All purified fractions were proven to contain hexuronic acid, although in variable proportions. A preliminary assessment, in terms of sulphate content, was performed by C-PAGE, confirming that sulphation degree increased together with the ionic strength of the buffer used for elution. Additionally, cytocompatibility of all sponge fractions was evaluated on a human cell line, showing no remarkable negative effects on cell viability, thus suggesting that these glycans did not interfere with essential metabolic pathways. Further experiments will be carried out to assess the full biocompatibility of these acidic glycans in vivo.

Moreover, the ability to inhibit the coagulation process was evaluated for each of the four sponge fractions, and the results indicated that the third and fourth hexuronate-containing fractions were able to strongly interfere with the intrinsic pathway of the coagulation cascade, showing effects comparable to the activities obtained for heparin (Hep) and enoxaparin (E-Hep). The same fractions also showed inhibitory activity, albeit to a lesser extent, on the extrinsic pathway. In this context, our results can have a valuable impact, as sulphated polysaccharides from marine invertebrates have been shown to possess beneficial and adjustable anticoagulant activity, even though with a lower activity with respect to Hep.

Both FT-IR and NMR analyses performed on the Ss3 fraction suggested that, by comparing obtained spectra with those from Ht3 and with data reported in the literature, the most abundant polysaccharide contained hexuronic acid, N-Acetylgalactosamine, and fucose, indicative of a fucosylated chondroitin sulphate.

Altogether, the results presented in this paper demonstrate, for the first time, the presence of acidic glycans with anticoagulant activity in the ECM of keratose sponges belonging to the genus *Sarcotragus*, whose structure showed similarities with fucosylated chondroitin sulphate extracted from sea cucumbers’ body wall. Further investigations will be carried out, aiming at confirming the obtained results, performing more detailed structural analyses, and assessing the clear structure–activity relationship of the sponge acidic polysaccharides, in addition to evaluating which coagulation factors are inhibited by these glycans.

The great diversity and metabolite complexity of marine sponges, as a key renewable source in sustainable shallow water plants, could represent a useful tool for applications in bioinspired bioactive compound science and technologies. The present findings suggest that the fucosylated chondroitin sulphate fraction purified from *S. spinosulus* could be a new anticoagulant drug candidate, useful as a substitute for currently adopted antithrombotics.

## Figures and Tables

**Table 1:** Yield of each purified fraction based on the weight of dehydrated delipidated tissue (DDT) from *Sarcotragus spinosulus* (Ss) and *Holothuria tubulosa* (Ht). Data are reported as mean ± standard deviation (* *n* = 18, ^§^ *n* = 7).

Fraction	Total Yield (mg UA/g DDT)	Yield (mg UA/g DDT)	Fraction % over Total Glycans Purified
Ss1 *	2.234	0.897 ± 0.018	40.15
Ss2 *		0.289 ± 0.011	12.94
Ss3 *		0.978 ± 0.072	43.78
Ss4 *		0.070 ± 0.005	3.13
Ht1 ^§^	5.068	0.332 ± 0.010	6.55
Ht2 ^§^		0.226 ± 0.017	4.46
Ht3 ^§^		4.400 ± 0.115	86.82
Ht4 ^§^		0.110 ± 0.010	2.17

## Data Availability

The data presented in this study are available on request from the corresponding authors.
